# Supplementary material for: Young dictators—Speaking about oneself decreases generosity in children from two cultural contexts
Source: PLoS One. 2024 Mar 7;19(3):e0300200. doi: 10.1371/journal.pone.0300200 (PMC10919844; doi:10.1371/journal.pone.0300200)
Supplement: S1 File — (DOCX) [file pone.0300200.s001.docx]

**Supporting Information S1**

This file contains the experimental instructions, as well as the full priming script.

**Experimental Instructions**

Sticker allocation

*Each testing session began by presenting the child with a selection of 10 colourful, animal stickers.*

“Do you like stickers? I‘ve got 10 stickers here, and you get to choose 6 of them that you can take home with you. Which ones do you want to choose? OK great. These are yours, but for now, we will leave them here on the table” (*The experimenter moves the stickers out of reach on table*).

*Next, each child took part in one of three priming-conditions in the form of semi-structured interviews. The interviews were designed to take approximately 4 minutes to complete. A watch was used to ensure that this time limit was held as closely as possible (i.e., asking the child to elaborate if necessary/appropriate).*

Self-focus interview:

“Firstly, I am going to ask you some questions. In my job, I am interested in how children talk about themselves. So I want you to think about yourself, and I am going to ask you lots of questions about you”.

1. What are you good at?

Follow up questions (if needed) adapted to child’s answer.

- - - - OK, how long have you been doing that for?
      - Do you score a lot of goals? (i.e., if child talks about being good a football player).

2. Do you have a favourite toy?

- - - - Have you had it for a long time?

3. What do you like to do when you are at home?

4. Can you tell me about something you can do independently (without anyone else)?

5. What do you like to do when you are completely alone?

6. What makes you special (unique)?

7. What is your favourite subject in school?

- - - - Why do you like it so much?

8. What do you want to be when you grow up?

- - - - Why?

9. How are you different from other people?

- - - - Is there maybe something that you really like, that other people might not like, or something that you are good at, that other people might not be so good at?

10. What are you most proud of?

- - - - When have you been proud of yourself?

Other-focus interview:

“Firstly, I am going to ask you some questions. In my job, I am interested in how children talk about their relationships with others. So I’m going to ask you lots of questions about your relationships with your friends and family”.

1. Is there anyone in your life that you feel close to (have a close connection with)?

- - - - Why do you think the two of you have such a close relationship?
      - Is there anyone else that you have a close connection with?

2. Do you have a big family?

- Are you close with your family?

3. Why is it important to have a family?

4. Do you have a lot of friends?

5. Do you have a best friend?

- What is the name of your best friend?

6. What is it that makes the two of you such good friend?

- What do you like to do together?

7. Why is it important to have good friends?

8. Can you tell me about a time you had to cooperate (work together) with someone else?

9. Is there anyone in your life that you depend on (or feel that you need)?

- Why do you depend on/need ___?

10. Are you part of a group, or a club, or a team?

- Why do you think it’s nice to be part of a group/club/team?

Control condition:

“Firstly, I am going to ask you some questions. In my job, I am interested in how children describe different things. Today I want you to think about different animals. Can you please list *all* the animals you can think of?”

“OK, great. One of the animals you mentioned was [a dog]. Can you please describe a dog for me in as much detail as you can?”

*The experimenter moved on to the next animal until approximately 4 minutes had passed.*

Dictator Game

*Following the interview, the experimenter stated:*

“Thank you for answering my questions. Now let’s look at these envelopes. I have two envelopes here. This envelope is yours. Everything you put in to this envelope is yours and you get to take it home with you. Let’s write your name on it so that we remember that you get to keep everything in it”.

*The child wrote his or her name on the envelope.*

“This other envelope is for another child. We don’t know the name of this child. All we know is that it is another child from a different school that I will visit next week. Everything that goes into this envelope is for that child and the child gets to take it home”.

“Now, if you want to, you can give some of your stickers to the other child. You don’t have to give any away. But if you want to, you can. If you do want to give any away, then you can put them into the other child’s envelope and post it into this box”.

*The experimenter placed an opaque post-bow on the table.*

“No-one will know which envelope came from you. The stickers you want to keep you can put into your envelope”.

“Just to check that you understand the game, I’ll ask you some questions.

- Do you have to share any stickers?

- Where will you put the stickers you want to keep?

- Which envelope belongs to the other child?

I will turn around and close my eyes so I cannot see what you do with the stickers. It will be your secret. When you’re done, just put the other child’s envelope into the box and let me know.”

*After a few minutes, the experimenter asked if the child had finished. If the child confirmed this, the experimenter informed the child that the task was over and thanked him/her for taking part. In addition to the remaining stickers, each child also received a small prize for taking part.*
