# Supplementary material for: Young dictators—Speaking about oneself decreases generosity in children from two cultural contexts
Source: PLoS One. 2024 Mar 7;19(3):e0300200. doi: 10.1371/journal.pone.0300200 (PMC10919844; doi:10.1371/journal.pone.0300200)
Supplement: S3 File — (DOCX) [file pone.0300200.s003.docx]

**Supporting Information S3**

This file contains summary statistics of all priming time data.

|  | **Self-focus** | **Other-focus** | **Control** | **Total** |
| --- | --- | --- | --- | --- |
| **India**  [95% CI] | 232.33  [228.28, 236.39] | 231.50  [227.31, 235.69] | 228.93  [225.86, 232.01] | 230.92  [228.79, 233.05] |
| **UK**  [95% CI] | 234.52  [228.08, 240.99] | 231.63  [227.43, 235.84] | 233.67  [229.71, 237.62] | 233.28  [230.49, 236.06] |

Table showing mean interview times (in seconds) and 95% confidence intervals (CI) for India and the UK, and across interview conditions in each culture.
